# Downregulation of Circ-PITHD1 Suppressed Colorectal Cancer via Glycolysis Inhibition through miR-590-5p/HK2 Axis

**DOI:** 10.1155/2022/7696841

**Published:** 2022-10-14

**Authors:** Shiguang Yang, Kui Zhao, Xiaodong Yang, Chungen Xing

**Affiliations:** Department of Gastrointestinal Surgery, The Second Affiliated Hospital of Soochow University, Sanxiang Road, Suzhou 215000, Jiangsu, China

## Abstract

Colorectal cancer (CRC) is a frequent malignancy around the globe. Circular RNAs (circRNAs) are implicated in CRC development. Nevertheless, the regulatory mechanisms and biological functions regarding circRNAs in CRC progression are largely unclear. The present investigation employed next-generation sequencing (NGS) to study the abnormal circRNA expression in CRC tissues. The regulatory mechanism and targets were then analyzed by bioinformatics, luciferase reporter analysis, CCK8, colony formation, and Transwell migration. *In vivo* metastasis and tumorigenesis assays were conducted to elucidate circ-PITHD1 roles regarding CRC. The data showed that circ-PITHD1 expression increased in a CRC cell line and tissues, which indicated that circ-PITHD1 functioned in CRC progression. circ-PITHD1 downregulation inhibited CRC invasion and proliferation in the experiments. Luciferase reporter results confirmed that both miR-590-5p and hexokinase 2 (HK2) were circ-PITHD1 downstream targets. HK2 overexpression or miR-590-5p suppression reversed CRC cell proliferation and invasion after silencing of circ-PITHD1 by regulation of glycolysis. Taken together, this investigation discovered that circ-PITHD1 downregulation suppressed CRC progression by inhibiting glycolysis via the miR-590-5p/HK2 axis.

## 1. Introduction

Colorectal cancer (CRC) is the 3rd most general tumor and the 4th most deadly cancer on the globe. ∼10% of all new cancer patients are CRC [1, 2]. Though neoadjuvant chemoradiotherapy, postoperative chemoradiotherapy, surgery, and immunotherapy are broadly utilized and are rapidly gaining acceptance among CRC patients, the prognosis of patients with advanced CRC remains poor [3]. The specific mechanism of CRC occurrence and development is still unclear, which is a major reason for the poor prognosis of CRC. Thus, it is urgent to identify CRC progression pathogenesis and develop special diagnostic biomarkers along with precise therapy targets.

Circular RNA (circRNA) is initiated due to pre-mRNAback-splicing [4]. Exonic circRNAs are the most common circRNAs, which are primarily localized in the cytoplasm to act with microRNAs (miRNAs) or RNA-binding proteins [5, 6]. An accumulation study found that circRNAs have important functions in various cancers. As a miRNA sponge, circRNAs regulate mRNA expression and ultimately improve their functions in cancer cell proliferation and metastasis [7, 8]. Previous investigations discovered that circ-0087862 promoted CRC progression by upregulating BACH1 expression via miR-142-3p sponging [9]. The expression of circ-0000212 promotes CRC cell proliferation by modulating FOXP4 expression via sponging miR-491 [10]. The role of circRNA in CRC progression is still unknown.

The present investigation sought to determine circRNA expression in CRC and to identify underlying mechanisms. The data showed that circ-PITHD1 is highly expressed in CRC cell lines, which increased CRC cell invasion and proliferation. Additionally, we found that circ-PITHD1 knockdown repressed CRC progression by inhibiting hexokinase 2 (HK2)-mediated glycolysis through sponging miR-590-5p. These data demonstrate that circ-PITHD1 may represent a novel CRC diagnosis and treatment target.

## 2. Materials and Methods

### 2.1. Patients

In total, we obtained six paired CRC samples and adjacent normal tissues from the Second Affiliated Hospital of Soochow University. The Ethics Committee in the Second Affiliated Hospital of Soochow University approved our study after receiving written consent from the patients. Tissues were stored at −80°C.

### 2.2. RNA Sequencing, Quantification, and Identifications

Total RNA was obtained from the freshly frozen CRC and adjacent tissue pairs. We used an Agilent 2200 system (Agilent Technologies, USA) to confirm the quality of the RNA. A RiboMinus eukaryote kit (QIAGEN, Valencia, CA, USA) was used to eliminate ribosomal RNA. We performed NGS with the Illumina HiSeq 3000 (Illumina, San Diego, CA, USA) and aligned reads. We collected unmapped reads to characterize circRNAs. We counted reads that aligned to circRNA junctions having an overhang of ≥6 nt for each candidate.

### 2.3. Fluorescence *In Situ* Hybridization

Specific probes for circ-PITHD1 (Dig-5′-CTTGCCAGACTTAAGCTTTTTACGACGCG-3′-Dig) were prepared (Geneseed Biotech, Guangzhou, China). The signals were captured by Cy3-conjugated antibiotin antibodies (Jackson Immuno Research Inc., West Grove, PA, USA). 4,6-Diamidino-2-phenylindole (DAPI) was employed for cell nuclei counterstaining. We detected images with the Zeiss LSM 700 confocal microscope (Carl Zeiss, Oberkochen, Germany).

### 2.4. Cell Culture

The CRC cell lines SW480, SW620, HT29, HCT116, and Lovo and normal human colonic epithelial cells NCM460 were cultivated in DMEM medium (Gibco, NY, USA) supplemented with 10% FBS (Gibco). The cells were then incubated in a humidified atmosphere. Subsequently, SW480 and Lovo cells were transfected with 50 nM of miR-590-5p inhibitor (Shanghai GenePharma Co., Ltd.), small interfering RNA (si) against circ-PITHD1 (si-circ-PITHD1, 5′-CGCUCCAAGUUUGUUGAAAUU-3′ (Shanghai GenePharma Co., Ltd.)), and the HK2 overexpression vector (HK2, HK2 cDNA sequence clone into pcDNA3.1 vector (Shanghai GenePharma Co., Ltd.)) via Lipofectamine 2000 (Invitrogen; Thermo Fisher Scientific, Inc.). SW480 and Lovo cells were collected 2 days after transfection. To better verify the circ-PITHD effects during the experiments, lentiviral-based small hairpin RNA (shRNA) targeting circ-PITHD1 was performed. The GFP process was made 3 d after infection into SW480, which had a green fluorescence >95%. For *in vivo* metastatic detection, pLVX-Luc2-P2A-AcGFP1-puro lentiviral vector (Novagen) was utilized for SW480 infection, which produced the luc-SW480 cell line.

### 2.5. Cell Transfection

GenePharma (Shanghai, China) provided the transfection plasmids. We preseeded cells into 6-well plates. Cell transfection was made following Lipofectamine 2000 protocols at 70% confluence. After 2 days, the transfected cells were harvested for subsequent experiments.

### 2.6. Quantitative Real-Time Polymerase Chain Reaction (qPCR)

We obtained total RNA from cells or tumor tissues utilizing the TRIzol reagent kit (Invitrogen, Carlsbad, CA, USA). cDNA was then synthesized and amplified by applying TaqMan miRNA reverse transcription kit. We made RT-qPCR by the TaqMan™ MicroRNA Assay Kit (^#^4440885, Applied Biosystems, Foster City, CA, USA). Finally, the 2^−ΔΔCT^ approach was used to capture the relative fold change of RNA expression. *U6* and *GAPDH* were utilized as internal references. Primers for the circ-PITHD1 expression assay included forward, 5′-CCTAATAAATCCTTGC-3′; reverse, 5′-CAGCTCCGGCAACTAAGCGCGC-3′.*miR-590-5p* primers: forward, 5′-GAGCTTATTCATAAAAGT-3′; reverse, 5′-TCCACGACACGCACTGGATACGAC-3′.*HK2* primers: forward, 5′-GAGCCACCACTCACCCTACT-3′; reverse, 5′-CCAGGCATTCGGCAATGTG-3′; U6 primers: forward, 5′-CTCGCTTCGGCAGCACA-3′; reverse: 5′-AACGCTTCACGAATTTGCGT-3′; GAPDH primers: forward, 5′-AATGGGCAGCCGTTAGGAAA-3′; reverse: 5′-TGAAGGGGTCATTGATGGCA-3′.

### 2.7. Cell Proliferations

The technician seeded cells into 96-well plates with 2 × 10^3^ cells/well density. At established time points, each sample absorbance was determined at 450 nm by applying the CCK-8 assay (Yeasen Biotech Co., Ltd, Shanghai, China). A cell viability curve was then plotted.

### 2.8. Transwell Migration Assay

A cell suspension of 2.0 × 10^5^/mL was added to each well (200 *μ*L/well) in a Transwell chamber (Millipore, Billerica, MA, USA) on the upper side. The 500 *μ*L medium, including 10% FBS, was put on the lower side. After 1 d incubation, migrated cells were fixed to the bottom side by 4% paraformaldehyde for 15 min before staining with 0.1% crystal violet for 5 min. We observed and calculated the number of migrated cells using a microscope. Six fields of view were randomly selected for each sample.

### 2.9. Lactate Production and Glucose Uptake Assay

Lovo cells together with SW480 cells were cultured in a glucose-free DMEM medium for 16 h. The medium was changed by high-glucose DMEM medium and the CRC cells were cultivated for another day. Lactate production and glucose uptake were determined by utilizing a lactate oxidase-based colorimetric assay and a fluorescence-based glucose assay kit (BioVision, Milpitas, CA, USA), respectively.

### 2.10. Dual-Luciferase Reporter Assay

Putative miR-590-5p binding site in HK2 and circ-PITHD1 (WT or MUT) 3′-UTR was cloned intopsi-CHECK (Promega, Madison, WI, USA) vector with firefly luciferase 3′-UTR or circ-PITHD1 as primary luciferase signals with Renilla luciferase as normalized signals, which we then called HK2-Wt/circ-PITHD1-Wt, and HK2-Mut/circ-PITHD1-Mut.psi-CHECK vector provided Renilla luciferase signal as normalization to compensate for differences between harvested efficiencies and transfections. HEK293-cell transfection was achieved employing Lipofectamine 2000 (Invitrogen Life Technologies, Carlsbad, CA, USA). Renilla and firefly luciferase activities were measured 24 h after transfections employing the dual-luciferase reporter assay system (Promega, Mannheim, Germany) and luminometer (Molecular Devices, USA). Relative Renilla luciferase activities were analyzed using Promega protocols (Mannheim, Germany).

### 2.11. *In Vivo* Experiments

CRC nude mouse models were prepared by injecting SW480 cells with sh-NC or sh-circ-PITHD1 into the mice flank. Tumor weight and volume were measured. The Animal Ethics Committee of the Second Affiliated Hospital of Soochow University approved experiments. The present research followed the Guide for Care and Use of Laboratory Animals.

For tumor metastasis analysis, stably transfected luminescence-labeled SW480 cells with sh-circ-PITHD1 or sh-NC, which we suspended in sterile PBS, were injected into every 4-week-old male nude mouse tail vein. After 4 w, the *in vivo* bioluminescence imaging system was used for lung metastasis evaluation. Metastatic foci numbers within lung tissues were measured following HE staining.

### 2.12. Statistical Analyses

Data are denoted by means ± SD. Statistics analysis was made utilizing GraphPad Prism (La Jolla, USA) to compare significance between groups. *P* value ≤0.05 was considered as statistically significance. Two-tailed Student's *t*-tests were utilized to calculate significant differences between groups. One-way ANOVA with post hoc Bonferroni tests was applied to compare significant differences between the groups.

## 3. Results

### 3.1. Circ-PITHD1 Functions Importantly for CRC Progression

**Figure 1:**
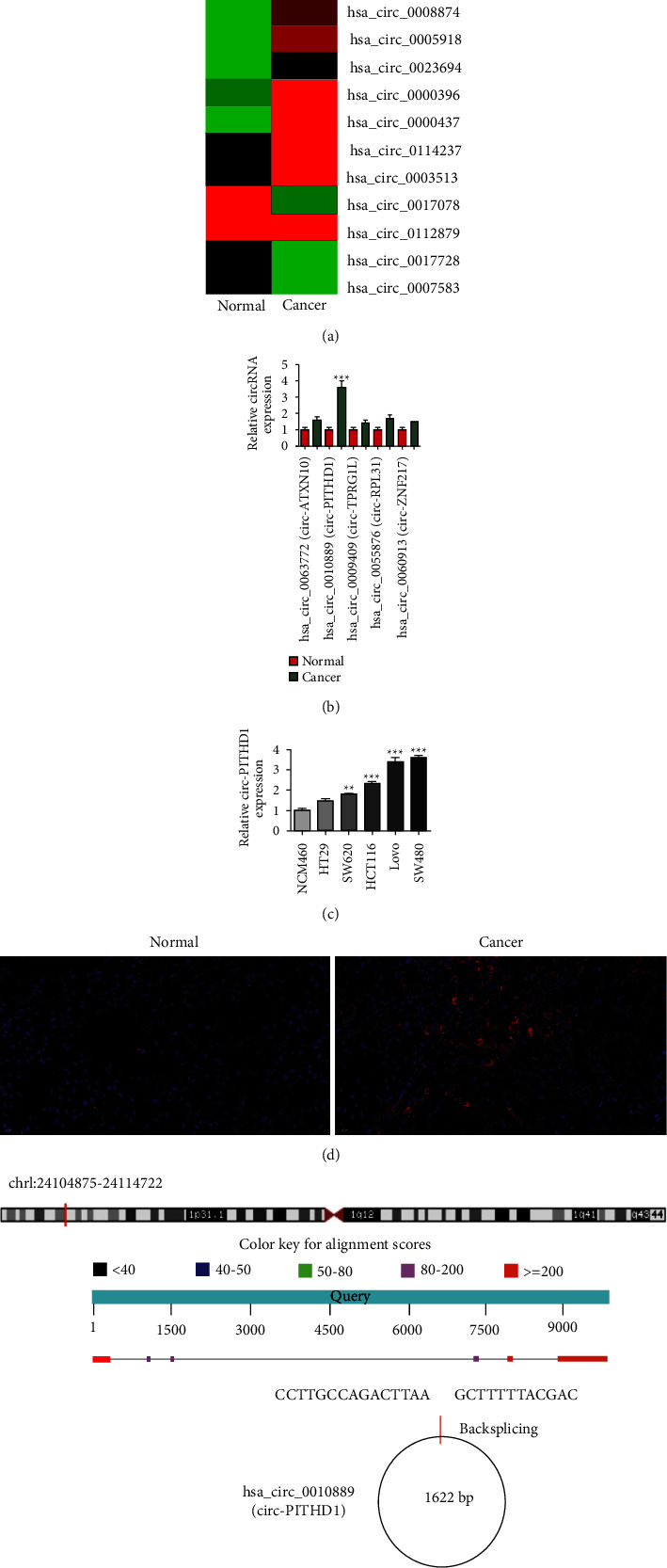
Circ-PITHD1 plays an important role in the progression of colorectal cancer. (a) Heat map showing the expression of circRNA in CRC tumor tissues and adjacent normal tissues. (b) RT-qPCR detection shows the expression of 5 high-expression circRNA in CRC tumor tissues and adjacent normal tissues. The data are presented as the mean ± SD. ^*∗∗∗*^*P* <  0.001 vs. normal. (c) RT-qPCR detection shows the expression of circ-PITHD1 in CRC cancer cell lines HT29, SW620, HCT116, Lovo, and SW480 and human normal colonic epithelial NCM460 cells. The data are presented as the mean ± SD. ^*∗∗*^*P* <  0.01, ^*∗∗∗*^*P* < 0.001 vs. NCM460. (d) FISH detection shows the expression and subcellular distribution of circ-PITHD1. (e) The genomic loci of the *PITHD1* gene and circ-PITHD1.

More and more studies have reported that circRNA has important functions in CRC progression [11], while the regulatory mechanism is unknown. The present investigation employed NGS and found that circRNA was abnormally expressed in CRC tissues compared with adjacent normal tissues (Figure 1(a)). RT-qPCR data revealed that five highly expressed circRNAs were observed according to NGS results. The data showcased that only circ-PITHD1 expression was upregulated significantly in CRC tissues (Figure 1(b)). The RT-qPCR data verified that circ-PITHD1 expression was increased in CRC cells, including SW480, SW620, and Lovo compared with normal human colonic epithelial NCM460 cells. Additionally, Lovo and SW480 cells had higher circ-PITHD1 expression (Figure 1(c)). FISH detection showcased that circ-PITHD1 expression increased in CRC tumor tissues compared to adjacent normal tissues. The outcomes confirmed that circ-PITHD1 was mainly distributed in the cytoplasm (Figure 1(d)). The circ-PITHD1 originated from cyclizing six exons of PITHD1, which are located at chr1:24104875–24114722. PITHD1 is 9847 bp and spliced mature circRNA is 1622 bp (Figure 1(e)). So our team termed hsa_circ_0010889 as circ-PITHD1.

### 3.2. The Circ-PITHD1 Downregulation Suppressed CRC Proliferation and Tumor Growth

**Figure 2:**
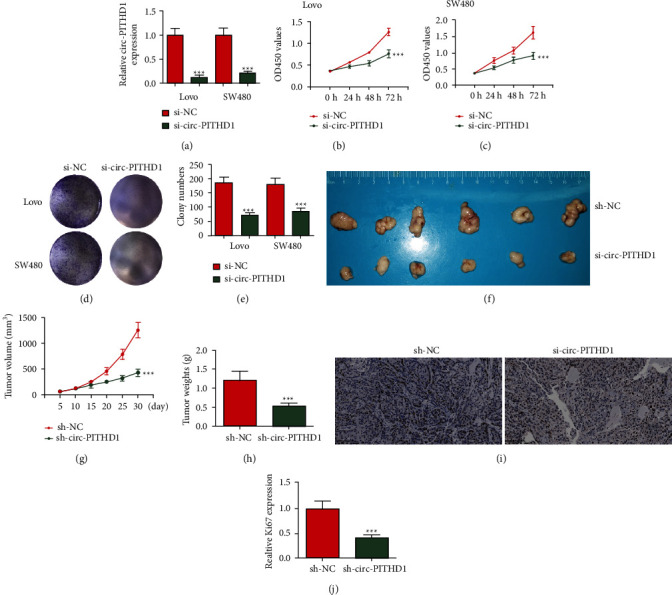
Downregulation circ-PITHD1 suppressed CRC proliferation and tumor growth in both *in vivo* and *in vitro* experiments. (a) RT-qPCR detection shows the expression of circ-PITHD1 in both Lovo and SW480. The data are presented as the mean ± SD. ^*∗∗∗*^*P* < 0.001 vs. si-NC. (b, c) CCK8 detection shows the effect of circ-PITHD1 on CRC cell proliferation. The data are presented as the mean ± SD. ^*∗∗∗*^*P* < 0.001 vs. si-NC. (d, e) Cloning formation assay showing the cell proliferation in both Lovo and SW480 cells. Data are presented as the mean ± SD. ^*∗∗∗*^*P* < 0.001 vs. si-NC. (f) Representative photographs of SW480 tumor formation in xenografts of nude mice. (g, h) Summary of tumor volumes and weight in mice. Data are presented as the mean ± SD. ^*∗∗∗*^*P* < 0.001 vs. sh-NC. (i, j) Immunohistochemistry shows the percentage of Ki-67-positive cells. The relative Ki-67-positive cells were calculated. Data are presented as the mean ± SD. ^*∗∗∗*^*P* < 0.001 vs. sh-NC.

To reveal the role of circ-PITHD1 in CRC progression, the technician prepared siRNA against circ-PITHD1 (si-circ-PITHD1), which was transfected into both SW480 and Lovo cells. Outputs showcased that circ-PITHD1 was significantly decreased after silencing circ-PITHD1 in both Lovo and SW480 cells (Figure 2). CCK8 (Figures 2(b)-2(c)) and cloning formation (Figures 2(d)-2(e)) assays confirmed that circ-PITHD1 downregulation significantly decreased proliferation of both Lovo and SW480 cells. The tumor formation in nude mouse xenografts following injection of SW480 cells showcased that circ-PITHD1 silencing significantly decreased tumor growth (Figures 2(f)–2(h)). Immunohistochemistry with Ki67 staining also confirmed that circ-PITHD1 silencing inhibited Ki67 expression in tumor tissues. The outcome data suggested that circ-PITHD1 downregulations inhibited CRC proliferation and tumor growth.

### 3.3. The Circ-PITHD1 Downregulation Suppressed CRC Migration and Pulmonary Metastasis

**Figure 3:**
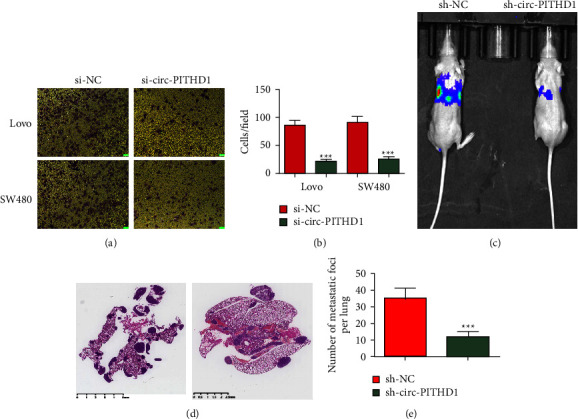
Downregulation of circ-PITHD1 suppressed CRC migration and pulmonary metastasis in both *in vivo* and *in vitro* experiments. (a) Transwell detection shows the migration of both Lovo and SW480 cells after transfected with si-circ-PITHD1. Data are presented as the mean ± SD. ^*∗∗∗*^*P* < 0.001 vs. si-NC. (c) Living imaging detection shows the SW480 cells' pulmonary metastasis. (d, e) The numbers of metastatic foci in lung tissues were calculated according to the HE staining. The data are expressed as the mean ± SD. ^*∗∗∗*^*P* < 0.001 vs. NC.

A Transwell migration study showcased that circ-PITHD1 silencing suppressed SW480 and Lovo cell migration (Figures 3(a)-3(b)). Living imaging showed pulmonary SW480 cell metastasis and circ-PITHD1 silencing decreased pulmonary metastasis capability by reducing metastatic foci numbers in lung tissues as shown by HE staining (Figures 3(c)–3(e)). These findings inferred that circ-PITHD1 downregulation inhibited CRC cell invasions.

### 3.4. The miR-590-5p and HK2 Were Circ-PITHD1 Downstream Targets

**Figure 4:**
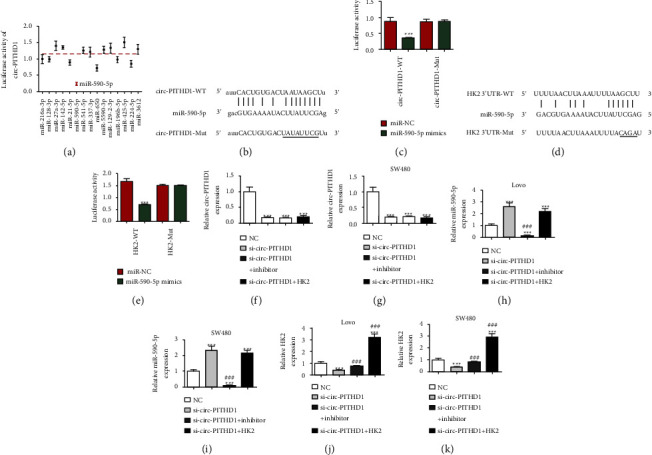
Both miR-590-5p and HK2 were the downstream targets of circ-PITHD1. (a) The luciferase activity of circ-PITHD1 in HEK293T cells transfected with different miRNA mimics, which are putative binding sites for the circ-PITHD1 sequence. Luciferase activity was normalized by Renilla luciferase activity. (b) Prediction of binding sites of miR-590-5p in circ-PITHD1. The MUT version of circ-PITHD1 is presented. (c) Relative luciferase activity determined 48 h after transfection of HEK293T cells with miR-590-5p mimic/NC or circ-PITHD1 WT/Mut. Data are presented as means ± SD. ^*∗∗∗*^*P* < 0.001. (d) Prediction of binding sites of miR-590-5p within the 3′UTR of HK2. The MUT version of 3′-UTR-HK2 is shown. (e) Relative luciferase activity determined 48 h after transfection of HEK293T cells with miR-590-5p mimic/NC or 3′UTR-HK2 WT/Mut. Data are presented as means ± SD. ^*∗∗∗*^*P* < 0.001. (f–k) RT-qPCR detection shows the expression of circ-PITHD1, miR-590-5p, and HK2 in both Lovo and SW480 after being transfected with si-circ-PITHD1, miR-590-5p inhibitor, and HK2 overexpression vector single or combined. Data are presented as means ± SD. ^*∗∗∗*^*P* < 0.001 vs. NC. ^###^*P* < 0.001 vs. si-circ-PITHD1.

Bioinformatics data showcased that circ-PITHD1 interacted with miRNAs such as miR-128-3p, miR-142-5p, miR-21-5p, and miR-541-5p. Luciferase reporter data including circ-PITHD1 sequence were present, which we transfected with miRNA mimics into HEK293 cells. Outputs showcased that miR-590-5p significantly reduced fluorescein intensity, which revealed that miR-590-5p was the circ-PITHD1 downstream target (Figure 4(a)). The luciferase reporter results verified that miR-590-5p suppressed luciferase activity in WT yet not MUT cells (Figures 4(b)-4(c)), indicating that miR-590-5p was the circ-PITHD1 target.

The results showed that HK2 was the miR-590-5p downstream target. To obtain correlations between miR-590-5p and HK2, we used WT or MUT 3′-UTR-HK2 sequences such as the miR-590-5p binding sequence in the luciferase reporter vector (Figure 4(d)). The technician transfected the luciferase reporter vector into HEK293 cells combined with or without the miR-590-5p mimic. Luciferase reporter results revealed that miR-590-5p suppressed luciferase activity in WT yet not MUT cells (Figure 4(e)) showing that HK2 was the miR-590-5p target.

RT-qPCR data illustrated that circ-PITHD1 expression decreased after siRNA transfection against circ-PITHD1 (si-circ-PITHD1). Nevertheless, the miR-590-5p inhibitor or HK2 overexpression cannot restore circ-PITHD1 level after si-circ-PITHD1 in either Lovo or SW480 cells (Figures 4(f)-4(g)), which indicated that miR-590-5p and HK2 were circ-PITHD1 downstream targets. RT-qPCR data unraveled that circ-PITHD1 silencing incremented miR-590-5p expression. HK2 overexpression did not affect si-circ-PITHD1-induced miR-590-5p expression (Figures 4(h)-4(i)) indicating that miR-590-5p was circ-PITHD1 downstream. Data informed that circ-PITHD1 silencing decremented HK2 expression, while miR-590-5p downregulation reversed the inhibitory effect regarding si-circ-PITHD1 for HK2 expressions. Following transfection with HK2 overexpression vector, HK2 expression significantly incremented (Figures 4(j)-4(k)) revealing that circ-PITHD1 enhanced HK2 expression by sponging miR-590-5p.

### 3.5. HK2 Overexpression or miR-590-5p Inhibition Reversed CRC Cell Proliferation and Invasion after Silencing of Circ-PITHD1

**Figure 5:**
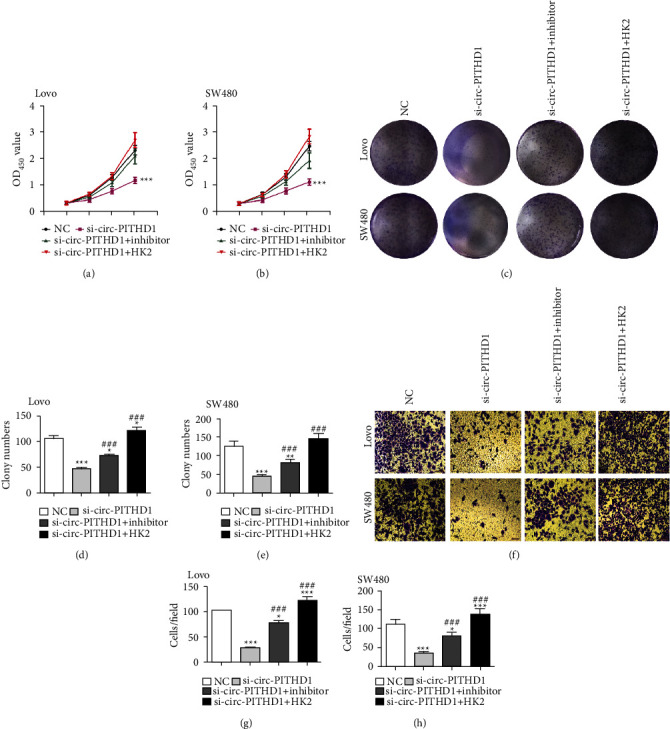
Overexpression of HK2 or inhibition of miR-590-5p reversed CRC cells proliferation and invasion after silencing circ-PITHD1. (a, b) CCK8 detection shows the proliferation ability of Lovo and SW480 cells. The data are expressed as the mean ± SD. ^*∗∗∗*^*P* < 0.001 vs. NC. ^###^*P* < 0.001 vs. si-circ-PITHD1. (c–e) Cloning formation assay shows the cell proliferation in both Lovo and SW480 cells. The data are expressed as the mean ± SD. ^*∗*^*P* < 0.05, ^*∗∗∗*^*P* < 0.001 vs. NC. ^###^*P* < 0.001 vs. si-circ-PITHD1. (f–h) Transwell detection shows the invasion and migration of Lovo and SW480 cells. The data are expressed as the mean ± SD. ^*∗*^*P* < 0.05, ^*∗∗∗*^*P* < 0.001 vs. NC. ^###^*P* < 0.001 vs. si-circ-PITHD1.

CCK8 (Figures 5(a)-5(b)) and cloning formation assays showcased that HK2 overexpression or miR-590-5p suppression reversed CRC cell proliferation in Lovo and SW480 cells after circ-PITHD1 silencing (Figures 5(c)–5(e)). The Transwell assay confirmed that HK2 overexpression or miR-590-5p suppression reversed CRC cell migration in Lovo and SW480 cells after circ-PITHD1 silencing (Figures 5(f)–5(h)).

### 3.6. HK2 Overexpression or miR-590-5p Inhibition Reversed CRC Cell Aerobic Glycolysis after Circ-PITHD1 Silencing

**Figure 6:**
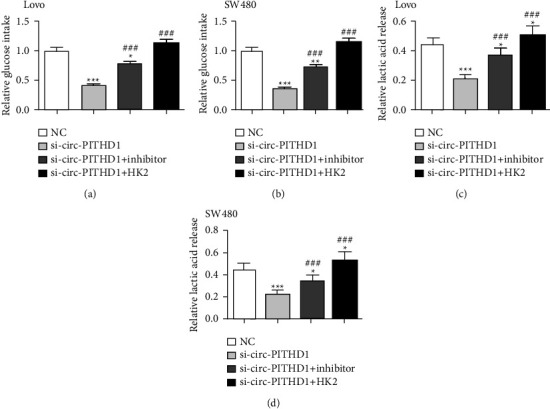
Overexpression of HK2 or inhibition of miR-590-5p reversed CRC cells aerobic glycolysis after silencing circ-PITHD1. (a–d) Glucose uptake (a, b) and lactate production (c, d) assays were employed to severally explain regulation of circ-PITHD1, miR-590-5p, and HK2 to glucose uptake and lactate production in both Lovo and SW480 cells. The data are expressed as the mean ± SD. ^*∗*^*P* < 0.05, ^*∗∗*^*P* < 0.01, ^*∗∗∗*^*P* < 0.001 vs. NC. ^###^*P* < 0.001 vs. si-circ-PITHD1.

The circ-PITHD1 silencing effects on the Warburg effect were analyzed. The data demonstrated that lactate production and glucose uptake decreased in the circ-PITHD1 silenced groups compared with the si-NC group. HK2 was found to be a dynamic metabolic enzyme in glycolysis, which can enhance glucose uptake. Thus, HK2 overexpression or miR-590-5p suppression reversed glucose uptake and lactate production after circ-PITHD1 silencing in Lovo and SW480 cells (Figures 6(a)–6(d)).

## 4. Discussion

Many investigations have reported that circRNAs have indispensable functions to regulate cancer progression, such as CRC [12]. For instance, circ-RNF121 regulates tumor progression along with glucose metabolism via the CRC miR-1224-5p/FOXM1 axis [13]. circRNA NOX4 enhances CRC development via the microRNA-485-5p/CKS1B axis [14]. The current investigation demonstrated that circ-PITHD1 expression is incremented in CRC cells and tissues, suggesting that circ-PITHD1 acts in CRC progression. circ-PITHD1 downregulation suppressed CRC invasion and proliferation. However, the regulatory mechanism is still largely unclear.

The bioinformatics analysis showed that HK2 and miR-590-5p were circ-PITHD1 downstream targets, which we verified via the luciferase reporter assay. circ-PITHD1 downregulation promoted miR-590-5p expression. miR-590-5p functions as an oncogene or tumor suppressor in renal carcinoma, cervical cancer, and osteosarcoma [15–17]. Previous investigations found that miR-590-5p expression was downregulated, but high miR-590-5p expression is associated with better CRC survival [18]. The miR-590-5p is an anti-onco-miR that suppresses CRC metastasis and angiogenesis via the NF90/VEGFA signaling axis [19]. This investigation demonstrated that miR-590-5p overexpression reversed si-circ-PITHD1 inhibitory effects upon CRC proliferation and migration. These findings indicate that circ-PITHD1 silencing inhibited CRC progression by promoting miR-590-5p expression.

Another study discovered that HK2 was the miR-590-5p downstream target, which was verified through a luciferase reporter assay. The circ-PITHD1 downregulation inhibited HK2 expression, while miR-590-5p suppression reversed inhibitory effects regarding si-circ-PITHD1 on HK2 expression. This study verified that HK2 had an important function in glycolytic metabolism regulation [20]. HK2 is a critical glycolytic enzyme catalyzing glucose conversion to glucose-6-phosphate, which is required for glycolysis. HK2 upregulation promoted cell invasion and migration by increasing glycolysis [21]. Aerobic glycolysis is also known as the Warburg Effect, which is a cancer hallmark. It is identified by changing its energy and glycolysis [22]. The current investigation also discovered that circ-PITHD1 silencing inhibited lactate production and glucose uptake, but HK2 overexpression restored inhibitory effects regarding si-circ-PITHD1 upon aerobic glycolysis. It is suggested that circ-PITHD1 silencing suppressed CRC progression via promoting miR-590-5p and inhibiting HK2 expression.

In conclusion, the current research provided proof that circ-PITHD1 downregulation decreased the invasion and proliferation ability of CRC by regulating miR-590-5p/HK2 signaling-mediated aerobic glycolysis. These findings show that circ-PITHD1 is a promising biomarker in CRC diagnostics, which will extend drug applications targeting circ-PITHD1 and indicates a role for circ-PITHD1 in CRC treatment.

## Data Availability

All data in this study are available from the corresponding author.
